# Effectiveness of a Multicomponent Treatment for Fibromyalgia Based on Pain Neuroscience Education, Exercise Therapy, Psychological Support, and Nature Exposure (NAT-FM): A Pragmatic Randomized Controlled Trial

**DOI:** 10.3390/jcm9103348

**Published:** 2020-10-18

**Authors:** Mayte Serrat, Míriam Almirall, Marta Musté, Juan P. Sanabria-Mazo, Albert Feliu-Soler, Jorge L. Méndez-Ulrich, Juan V. Luciano, Antoni Sanz

**Affiliations:** 1 Unitat d’Expertesa en Síndromes de Sensibilització Central, Servei de Reumatologia, Vall d’Hebron Hospital Universitari, Vall d’Hebron Barcelona Hospital Campus, Passeig Vall d’Hebron 119-129, 08035 Barcelona, Spain; mserrat@vhebron.net (M.S.); miriamalmirall1974@gmail.com (M.A.); mmuste@vhebron.net (M.M.); 2 Stress and Health Research Group, Departament de Psicologia Bàsica, Evolutiva i de l’Educació, Universitat Autònoma de Barcelona, 08193 Bellaterra, Spain; jp.sanabria@pssjd.org (J.P.S.-M.); a.feliu@pssjd.org (A.F.-S.); 3 Escola Universitària de Fisioteràpia, Escoles Universitàries Gimbernat, Universitat Autònoma de Barcelona, Sant Cugat del Vallès, 08174 Barcelona, Spain; 4 Institut de Recerca Sant Joan de Déu, Esplugues de Llobregat, 08950 Catalonia, Spain; 5 Teaching, Research, & Innovation Unit—Parc Sanitari Sant Joan de Déu, St. Boi de Llobregat, 08830 Catalonia, Spain; 6 Department of Medicine, International University of Catalonia, C/Josep Trueta s/n, Sant Cugat del Vallès, 08195 Barcelona, Spain; 7 Research Group on Socioeducative Interventions in Childhood and Youth (GRISIJ), Department of Methods of Research and Diagnosis in Education, Faculty on Education, University of Barcelona, 08007 Barcelona, Spain; 8 Sport Research Institute UAB, Universitat Autònoma de Barcelona, 08193 Bellaterra, Spain

**Keywords:** fibromyalgia, multicomponent treatment, pain neuroscience education, exercise therapy, cognitive behavioral therapy, mindfulness, nature exposure, randomized controlled trial

## Abstract

A recent study (FIBROWALK) has supported the effectiveness of a multicomponent treatment based on pain neuroscience education (PNE), exercise therapy (TE), cognitive behavioral therapy (CBT), and mindfulness in patients with fibromyalgia. The aim of the present RCT was: (a) to analyze the effectiveness of a 12-week multicomponent treatment (nature activity therapy for fibromyalgia, NAT-FM) based on the same therapeutic components described above plus nature exposure to maximize improvements in functional impairment (primary outcome), as well as pain, fatigue, anxiety-depression, physical functioning, positive and negative affect, self-esteem, and perceived stress (secondary outcomes), and kinesiophobia, pain catastrophizing thoughts, personal perceived competence, and cognitive emotion regulation (process variables) compared with treatment as usual (TAU); (b) to preliminarily assess the effects of the nature-based activities included (yoga, Nordic walking, nature photography, and Shinrin Yoku); and (c) to examine whether the positive effects of TAU + NAT-FM on primary and secondary outcomes at post-treatment were mediated through baseline to six-week changes in process variables. A total of 169 FM patients were randomized into two study arms: TAU + NAT-FM vs. TAU alone. Data were collected at baseline, at six-week of treatment, at post-treatment, and throughout treatment by ecological momentary assessment (EMA). Using an intention to treat (ITT) approach, linear mixed-effects models and mediational models through path analyses were computed. Overall, TAU + NAT-FM was significantly more effective than TAU at posttreatment for the primary and secondary outcomes evaluated, as well as for the process variables. Moderate-to-large effect sizes were achieved at six-weeks for functional impairment, anxiety, kinesiophobia, perceived competence, and positive reappraisal. The number needed to treat (NNT) was 3 (95%CI = 1.6–3.2). The nature activities yielded an improvement in affective valence, arousal, dominance, fatigue, pain, stress, and self-efficacy. Kinesiophobia and perceived competence were the mediators that could explain a significant part of the improvements obtained with TAU + NAT-FM treatment. TAU + NAT-FM is an effective co-adjuvant multicomponent treatment for improving FM-related symptoms.

## 1. Introduction

Fibromyalgia (FM) is a syndrome that affects around 2% of the general population [1] and has a strong impact on activities of daily living [2]. It usually affects women [3], but some recent studies have reported a prevalence of up to 40% in men due to the use of the 2016 American College of Rheumatology (ACR) diagnostic criteria [4,5]. High direct medical costs and indirect costs of this condition represent a great burden for the healthcare system of developed countries [6].

The etiopathogenesis of FM remains poorly understood, but the sensitization of the central nervous system (CNS) (i.e., central sensitization or CS) involving an imbalance between pain descending inhibitory and facilitatory pathways [3] appears to be involved in both the development and chronification of pain [7]. In the pathogenesis of central sensitization syndromes (SSC) such as FM, the phenomenon of CS is considered to be more relevant than that of peripheral sensitization, although it has been accepted that both can be involved [8]. CS is a broad concept involving a large variety of complex pathophysiological mechanisms [9,10] that represents a challenge for researchers and clinicians in the field of SSC.

The main clinical characteristics of FM include chronic widespread musculoskeletal pain, increased pain sensitivity incorporating allodynia and hyperalgesia, but without a known structural pathology in muscles, tendons, ligaments, or joints. It is usually accompanied by fatigue, sleep problems, paresthesia, joint stiffness, headache, cognitive problems, depression, and/or anxiety disorders [4,11].

Given that FM is a complex multidimensional disease that involves a variety of predisposing factors, a multicomponent approach is usually recommended, combining pharmacological and non-pharmacological treatments [12,13,14,15]. Empirical evidence supports the use of multicomponent treatments as the most beneficial interventions for FM patients and it has been claimed that they should be considered as the gold standard [16,17,18]. Regarding pharmacological treatment, the European League against Rheumatism (EULAR) recommendation for FM patients is to only use medication to control pain and sleep disturbances [19].

Use of the following therapeutic components have received empirical support and are considered to be optimal elements in multicomponent interventional packages: pain neuroscience education (PNE), exercise therapy (ET), cognitive behavioral therapy (CBT), and mindfulness training (MT) [20,21,22,23,24,25,26,27]. A recent randomized controlled trial (RCT) [27], which integrated, for the first time, the aforementioned four therapeutic approaches, PNE, ET, CBT and MT, found moderate to large effect sizes in the improvement of the core FM symptoms.

At the same time, therapeutic programs based on activities in nature have shown promise for improving mental health [28,29,30,31] in various clinical populations [32,33]. A growing number of studies suggest that exposure to nature directly produces effects in the affective (positive and negative emotions, and stress) and cognitive (attention, memory, etc.) domains, as well as can upward modulate the beneficial effects of physical activity. Likewise, it has been proposed that practice in a natural context could increase adherence to therapies based on the practice of physical activity (which has been called “green exercise”). These effects have been also tested in chronic pain patients [34] and those with FM [35]. However, to our knowledge, the synergistic effects of the therapeutic components mentioned above, and nature-based activities have not yet been tested in patients with FM.

Taking this gap in the literature as our starting point, the NAT-FM (acronym for nature activity therapy for FM) study aims to empirically test, for the first time, the impact of combining all the approaches described above, that is, integrating PNE, TE, CBT, MT, and exposure to natural contexts. NAT-FM is rooted on the body of scientific evidence reported in various studies [27,36], assumes a process-based therapy approach [37] and is expected to constitute an add-on therapy conceived as a new generation of therapeutic programs for FM, chronic pain in general, and various health problems, by combining different approaches in natural contexts.

The objective of this RCT was three-fold: (a) to analyze the effectiveness of a 12-week multicomponent treatment based on PNE, TE, CBT, MT, and nature exposure, as an add-on to treatment as usual (TAU) to improve functional impairment (primary outcome), as well as pain, fatigue, anxiety-depression, physical functioning, positive and negative affect, self-esteem, and perceived stress (secondary outcomes), kinesiophobia, pain catastrophizing thoughts, personal perceived competence, and cognitive emotion regulation (process variables) compared with TAU; (b) to preliminarily assess the specific effects of the nature activities included in TAU + NAT-FM; and (c) to examine whether the positive effects of TAU + NAT-FM on primary and secondary outcomes (post-treatment) were mediated through baseline to six-week changes in process variables.

## 2. Materials and Methods

### 2.1. Design

A detailed description of the study protocol is provided elsewhere [28]. A 12-week RCT was conducted in which the study participants were randomly allocated to 2 arms (using computer generated numbers): (a) TAU (control group) and (b) TAU + NAT-FM (active group). Data collection was conducted at baseline (pre), after week six of treatment (during), and after (post) treatment completion. Clinical assessments were conducted at pre-established time points: (a) classical structural assessment (CSA): pre, during and post-treatment; and (b) EMA: intrasession (nature activity log). Therefore, this study combines different types of assessments (CSA + EMA) to obtain more precise information about the temporal dynamics of the variables to be evaluated and, specifically, to record the affective and cognitive impact of each nature activity. Before the RCT, a proof of concept [38] of a simplified version of the study protocol was performed in order to assess its feasibility.

This study was conducted in accordance with the ethical standards set forth in the 1964 Declaration of Helsinki and its subsequent amendments. The hospital’s Ethics Committee of the University Hospital Vall d’Hebron (UHVH) evaluated and approved the study protocol (PR(AG)120/2018). This RCT was registered at ClinicalTrials.gov (NCT04190771) and we have followed the guidelines issued by the Consolidated Standards of Reporting Trials (CONSORT) [39].

### 2.2. Participants

**Figure 1:**
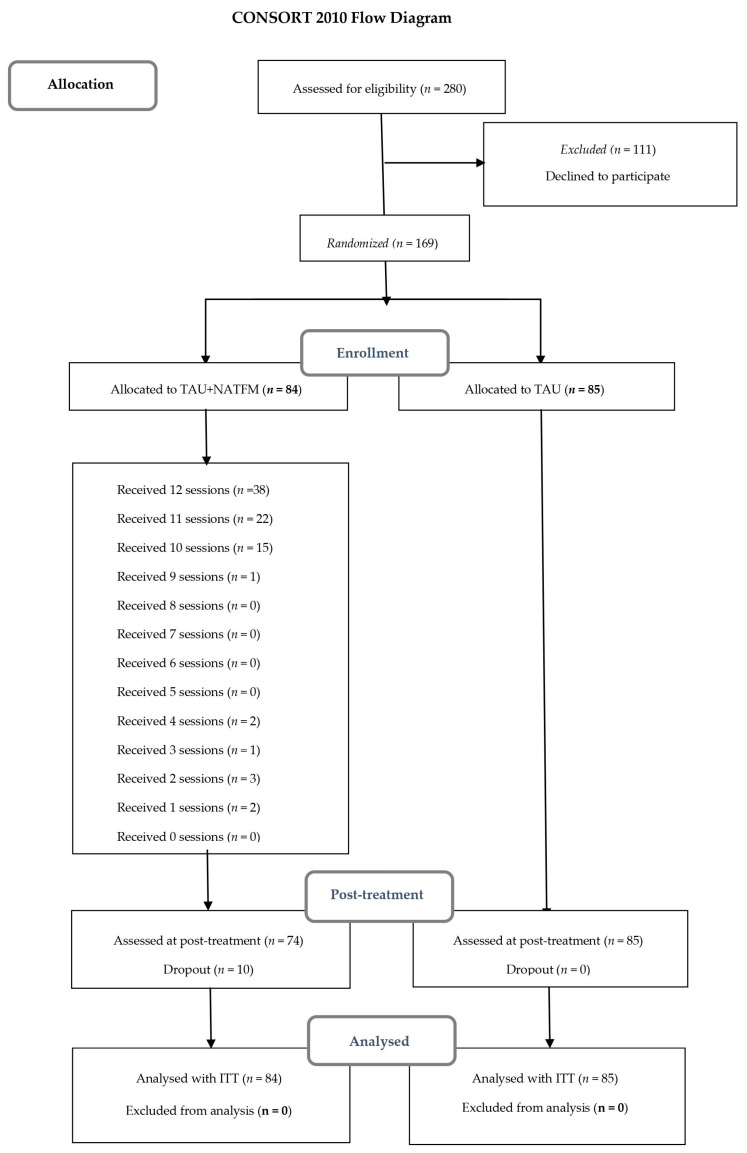
Flowchart of the nature activity therapy for fibromyalgia (NAT-FM) study following the Consolidated Standards of Reporting Trials (CONSORT) statement. ITT, intention-to-treat; *n* = 0 indicates that none of the participants attended the specified number of sessions.

A total of 169 out of 280 patients with FM who met the selection criteria were recruited by a physical therapist (M.S.) of the Central Sensitivity Syndromes Specialized Unit (CSSSU) at the UHVH from September to November 2019 and the patients participated in the TAU + NAT-FM study between November 2019 and February 2020. As shown in Figure 1, these patients were randomly allocated into two study arms, active group (*n* = 84), and a control group that received only TAU (*n* = 85). The active study arm included four waves of group-based therapy (approximately *n* = 21 per group), that is, all the groups did not receive the interventions and evaluations in the same period due to logistical reasons.

The inclusion criteria were: (a) being adults ≥ 18 years old, (b) meeting the 2010/2011 ACR diagnostic criteria for FM [4,40,41,42], and (c) agreeing to voluntarily participate in the study. Participants were excluded if: (a) they were already participants in concurrent or past (last year) RCTs and (b) they reported comorbidity with severe mental disorders or neurodegenerative diseases that would limit their capacity to participate in the study. Patients having a severe mental or neurodegenerative disorder that could make it difficult to follow up any type of group clinical session or physical activity, were not included in the list of candidates to be recruited. Once the list was defined according to this exclusion criterion, a second filtering was carried out excluding patients with a diagnosis of mental health disorder, who may have difficulties in participating in clinical sessions in a natural/outdoor context. This essentially includes patients diagnosed with agoraphobia. In this RCT, no participant was excluded due to this criterion. The CONSORT 2010 flow chart is shown in Figure 1.

### 2.3. Procedure

The procedure for recruiting the participants can be found in the study protocol [28]. In brief, all patients were consecutively screened by the physical therapist (M.S.) in the Central Sensitivity Syndromes Specialized Unit (CSSSU) at the UHVH in the context of clinical practice. Those participants who met the inclusion/exclusion criteria were scheduled for a face-to face interview with the principal researcher (M.S.) to provide an overview of the study. Those participants who were interested and agreed to participate signed a written informed consent. Each participant was then assigned to an alphanumeric code list using the Statistical Package for Social Sciences (IBM-SPSS v26, (Armonk, NY, USA) for random allocation to either TAU + NAT-FM or TAU.

### 2.4. Study Arms

#### 2.4.1. TAU + NAT-FM

The active group received PNE, TE, CBT, MT, and nature exposure. All the sessions were conducted outdoors. The TAU + NAT-FM protocol integrates the FIBROWALK protocol [27], replacing the exercise therapy described there for activities carried out in nature such as Yoga, Nordic walking, photography, and Shinrin Yoku. Table 1 and Table 2 provide an updated outline of the TAU + NAT-FM program described elsewhere [27,28].

The TAU + NAT-FM sessions were carried out once per week (2 h) for 12 consecutive weeks and were run in four groups arranged in a series. The sessions were directed by M.S., who is the physiotherapist of the CSSU of Vall d’Hebron University Hospital, and also a psychologist and sports technician with the required legal qualifications and extensive training for conducting the correct execution of this treatment. M.S. also had the help of a FM patient who had previously successfully completed the FIBROWALK program and joined the groups to explain her experience and to motivate them to maintain adherence to the therapy.

Patients of this intervention arm were requested for maintaining the prescribed drugs adjusted to their own symptomatic profile, and therefore to not change their medication regimen throughout the three-month period.

#### 2.4.2. TAU

In the present study usual care was based on basic education of the disease, advice on aerobic exercise and pharmacological treatment adjusted to the personal picture of each patient that basically consisted of duloxetine (30–60 mg/day) or amitriptyline (25 mg/day), pregabaline (150–300 mg/day) or tramadol at low doses, in monotherapy or combination... In the present study, we asked the patients to not change their medication regimen throughout the three-month period. For ethical reasons, we provided some complementary unstructured advice on PNE and aerobic exercise adapted to the physical capacities of the patients at the beginning of the study. Therefore, this might be regarded as slightly “enriched” usual care. Patients in the control group were placed on a waiting list to be recruited for the next TAU + NAT-FM treatment at the end of the present RCT (three months).

### 2.5. Outcome Measures

#### 2.5.1. Classical Structural Assessment (CSA) at Specific Time Points

##### Primary Outcome

The fibromyalgia impact questionnaire revised (FIQR) [43] was used to measure functional impairment during the last week. This instrument consists of three dimensions: physical dysfunction (scores from 0 to 30), overall impact (scores from 0 to 20), and intensity of the symptoms (scores from 0 to 50). Higher scores indicate greater impairment. The Spanish version shows adequate internal consistency (Cronbach’s α = 0.93) [44,45,46].

##### Secondary Outcomes

The visual analog scale (VAS) [43] was used to measure fatigue and pain, with scores ranging from 0 to 10. Higher scores are taken to indicate greater impairments due to fatigue and pain, respectively.

The hospital anxiety and depression scale (HADS) [47] was used to quantify the severity of anxiety and depression symptoms. It consists of two dimensions (anxiety and depression) of 7 items each, with a four-point Likert scale. Total scores of each scale, HADS-A and HADS-D, range from 0 to 21. Higher scores indicate greater symptom severity. The Spanish version shows adequate internal consistency for HADS-A (Cronbach’s α = 0.83) and for HADS-D (α = 0.87) [48].

The physical functioning component of the 36-item short form survey (SF-36) [49] was used to assess physical function. This dimension comprises a total of 10 items, with a three-point Likert scale. Total scores are transformed in order to range from 0 to 100. Higher scores indicate better physical functioning. The Spanish version shows adequate internal consistency (Cronbach’s α = 0.94) [50].

The positive affect and negative affect schedule (PANAS) [51] was used to evaluate affect. It has two dimensions (positive affect and negative affect) of 10 items each, answered on a 5-point Likert scale. Higher scores indicate a greater presence of specific affectivity. The Spanish version presents adequate internal consistency for PANAS-PA (Cronbach’s α = 0.92) and for PANAS-NA (Cronbach’s α = 0.88) [52].

The Rosenberg self-esteem scale (RSES) [53] was used to measure self-esteem. This instrument consists of 10 items that are answered on a four-point Likert scale. The total scores of each scale range from 10 to 40. Higher scores indicate higher self-esteem. The Spanish version has adequate internal consistency (Cronbach’s α = 0.87) and acceptable test-retest reliability (r = 0.72 to 0.74) [54].

The perceived stress scale (PSS-4) [55] was used to evaluate the perceived stress during the last month. In this study the short four-item version was employed, which is answered on a five-point Likert scale; therefore, total scores can vary from 0 to 16. Higher scores indicate higher perceived stress. The Spanish version shows acceptable internal consistency (Cronbach’s α = 0.77) [56].

##### Process Variables

The Tampa scale for kinesiophobia (TSK) [57] was used to measure fear of pain and movement. This scale consists of 11 items, which are answered on a four-point Likert scale. Total scores of each scale range from 11 to 44. Higher scores are taken to indicate greater fear of pain and movement. The Spanish version has adequate internal consistency (Cronbach’s α = 0.79) [58].

The pain catastrophizing scale (PCS) [59] was used to evaluate pain catastrophizing thoughts. This scale consists of three dimensions (rumination, magnification, and helplessness), with 13 items that are answered on a five-point Likert scale, and total scores ranging from 0 to 52. Higher scores indicate greater catastrophic thinking. The Spanish version shows adequate internal consistency (Cronbach’s α = 0.79) and acceptable test-retest reliability (r = 0.84) [60].

The personal perceived competence scale (PPCS) [61] was used to measure perceived competence. This scale consists of 8 items that are answered on a six-point Likert scale. Total scores of each scale range from 8 to 48. Higher scores indicate greater perceived competence. The Spanish version shows adequate internal consistency (Cronbach’s α = 0.83) [62].

The cognitive emotion regulation questionnaire (CERQ) [63] was used to assess individual differences in the cognitive regulation of emotions. This study used the short 18-item version. Responses were given on a five-point Likert scale (from 1 = almost never to 5 = almost always). Higher scores indicate higher frequency of use of each cognitive strategy. The Spanish version shows adequate internal consistency (Cronbach’s α*s* ranging from 0.77 to 0.93) and acceptable test-retest reliability (*r* ranging from 0.60 to 0.85) [64].

#### 2.5.2. Ecological Momentary Assessment (EMA)

The ecological momentary assessment (EMA) is a longitudinal, prospective research methodology that evaluates daily the variables of interest in real time and in a real context [65]. This methodology shows high reliability and validity [66], and in clinical research it is of special interest for the continuous assessment of the processes moderating the effectiveness of the interventions. It is also employed to evaluate the dynamics of the transfer and generalization of the effect of therapeutic sessions to the daily life of patients [67]. Its use as a complementary evaluation of psychological processes in clinical studies has recently increased thanks to the universalization of smartphones and apps. EMA was used to explore the effect of the different activities included in the TAU + NAT-FM treatment, assessing the specific short-term impact of each activity in terms of transferring the treatment effects to daily life. The intra-session assessments were conducted before and after each of the treatment sessions.

### 2.6. Statistical Analysis

In order to detect potential differences in sociodemographic and baseline clinical characteristics, we applied the *χ*^2^ test with continuity correction (or the two-sided Fisher exact test when appropriate) for categorical data and the student-Fisher *t*-test for continuous variables.

The main between-group analysis to assess the treatment effect was conducted on an intention-to-treat (ITT) basis, assuming data were missing at random. Linear mixed models were applied with maximum likelihood regression to account for the correlation between repeated measures for each individual. Multiple imputation has been reported to be unnecessary when computing linear mixed models [65]. Regression coefficients were calculated along with the respective 95% confidence intervals (95% CIs) for each group × time interaction. Effect sizes were reported using Cohen’s *d* for each pair comparison, using the pooled baseline SD to weight the differences in the pre-post mean values and to correct for the population estimate [66]. Separate models were estimated for each secondary outcome using the same strategy. The Benjamini–Hochberg correction test was applied to detect false discovery when performing multiple comparisons, since this test has been reported to overcome some limitations of other similar tests [66].

To further assess the clinical significance of improvements in the primary outcome (i.e., FIQR), we classified the sample into two categories: responders vs. non-responders to treatment. A responder is a patient that had decreased their FIQR posttreatment score by at least 20% in comparison with their baseline assessment [43]. This classification was later used to compute the number needed to treat (NNT), which refers to the estimated number of patients that should be treated with the new proposed intervention (i.e., TAU + NAT-FM) compared with the control group for obtaining 1 additional responder. The NNT was calculated along with a 95% CI.

To preliminarily assess the effect of the various activities included in TAU + NAT-FM (i.e., yoga, Nordic walking, nature photography, and Shinrin Yoku), paired-sample student-Fisher’s *t*-tests were conducted for each activity, analyzing the change produced in affective valence, arousal, dominance, fatigue, pain, stress and self-efficacy after practicing the activity.

Finally, we also examined whether the effects of TAU + NAT-FM on primary and secondary outcomes (post-treatment) were mediated through baseline to six-week changes in process variables (i.e., TSK, PCS, PPCS, and CERQ). We computed bivariate Pearson correlations between the baseline to six-week change in the process variables and the pretreatment to posttreatment change in the outcomes in order to detect potential significant relationships. In those cases in which more than two changes in process variables presented significant correlations with changes in clinical outcomes, only the two highest correlations were selected to ensure sufficient statistical power. We explored the direct and indirect associations between the treatment condition (TAU + NAT-FM vs. TAU as independent variable), TSK, PCS, PPCS, and CERQ subscales (mediators), and primary and secondary outcomes (dependent variables) using path analyses. Only data from participants with no missing data were used for this analysis (sample of completers). Regression coefficients (*B*) of bias-corrected bootstrapped indirect effects were calculated, as well as the corresponding SEs and 95% CIs [68].

Data analyses were computed using IBM-SPSS v26 and MPlus v7 (https://www.statmodel.com/). A 5% significance level was adopted in all two-tailed tests.

## 3. Results

### 3.1. Baseline Sociodemographic and Clinical Characteristics of the Groups

As can be seen in Table 3, no significant between-group differences were found in terms of sociodemographic variables (all *p* ≥ 0.08). The patients were female (99%), middle-aged (mean ± SD, 54 ± 9), and slightly overweight (body mass index, BMI: mean ± SD, 27 ± 6) and they were able to recall being ill for approximately 18 years. Most patients 59% were married or living with a partner, 63% lived accompanied, 50% had completed secondary studies, 50% were in paid employment, and 50% were processing some kind of disability certification at baseline. In relation to comorbidity, 85% had chronic fatigue, 32% multiple chemical sensitivity, 46% irritable bowel syndrome, and 58% migraines. Finally, 40% of the participants were taking more than two medications.

In the control intervention group (TAU + NAT-FM), there were 10 dropouts (11.7%), whilst in the control group (TAU) there were none. Therefore, only 5.9% of the total sample withdrawn from the study. When comparing baseline differences between dropouts and non-dropouts in terms of sociodemographic and clinical variables, non-statistical differences were found.

### 3.2. Effects on Functional Impairment (Primary Outcome)

Descriptive statistics and between-group analyses for the FIQR data as the primary outcome of this study are displayed in Table 4. In comparison with TAU, patients assigned to TAU + NAT-FM achieved a significantly greater reduction in functional impact, showing considerable effects after six weeks of treatment (d = 1.13) and at posttreatment (d = 1.83). These effects remained statistically significant after the Benjamini–Hochberg correction test was applied.

### 3.3. Effects on Pain, Fatigue, Anxiety-Depression, Physical Functioning, Positive and Negative Affect, Self-Esteem, and Perceived Stress (Secondary Outcomes)

Patients that received TAU + NAT-FM showed a significant reduction in self-reported pain compared with TAU. Moderate effects were found at 6 weeks (d = 0.66) and large effects at posttreatment (d = 5.62). Similar patterns were observed in depressive symptomatology after 6 weeks of treatment (d = 0.49) and at posttreatment (d = 1.45) and in physical functioning at 6 weeks (d = 0.53) and at posttreatment (d = 1.45). For the other secondary outcomes, a large effect of TAU + NAT-FM was found in comparison with TAU for fatigue at 6 weeks (d = 0.77) and at posttreatment (d = 0.93) and for anxiety at 6 weeks (d = 0.99) and at posttreatment (d = 1.59). The effects of all of these secondary outcomes remained statistically significant after the Benjamini–Hochberg correction test was applied. We found a small significant effect of TAU + NAT-FM compared with TAU for positive affect at posttreatment (d = 0.40). This effect became marginally significant after computing the Benjamini–Hochberg correction test. There were no statistically significant differences for the other variables or measurement times.

### 3.4. Effects on Kinesiophobia, Pain Catastrophizing, Personal Perceived Competence, and Cognitive Emotion Regulation (Process Variables)

Descriptive statistics and between-group analyses for the process variables are displayed in Table 5. TAU + NAT-FM achieved a significantly greater reduction than TAU for kinesiophobia, with large effects at both 6 weeks of treatment (d = 1.18) and at posttreatment (d = 2.20). The same patterns were observed in pain catastrophizing at 6 weeks (d = 1.21) and at posttreatment (d = 2.03), in personal perceived competence at 6 weeks (d = 0.72) and posttreatment (d = 1.20) and in positive reappraisal (CERQ) at 6 weeks (d = 0.72) and posttreatment (d = 1.42). Moderate effects of TAU + NAT-FM compared with TAU were found after 6 weeks of treatment for refocusing (CERQ) (d = 0.52) and planning (CERQ) (d = 0.58), finding large effects at posttreatment for the same outcomes, i.e., refocusing (CERQ) (d = 0.99) and planning (CERQ) (d = 0.83). For the other process variables, a small effect of TAU + NAT-FM was observed in comparison with NAT for acceptance (CERQ) at 6 weeks (d = 0.47) and moderate effects were found at posttreatment for both acceptance (CERQ) (d = 0.53) and perspective taking (CERQ) (d = 0.71). The effects of all of these secondary outcomes remained statistically significant after the Benjamini-Hochberg correction test was applied. A small effect of TAU + NAT-FM in comparison with TAU was found in catastrophizing (CERQ) at 6 weeks (d = 0.47) and at posttreatment (d = 0.85), although these latter effects became only marginally significant after applying the Benjamini–Hochberg correction test.

### 3.5. Number Needed to Treat

Thirty-eight patients in the TAU + NAT-FM group (66.7%) reached the status of responder (i.e., showed a decrease in their FIQR total score by at least 20% in comparison with baseline assessment), whilst only 15 patients in the TAU condition (20%) achieved this status. One patient (1.3%) from the active group showed a reduction of more than 70% in the FIQR scale and a total of 6 patients (8.1%) from this group showed a reduction of more than 60%.

The absolute risk reduction (ARR) in TAU + NAT-FM in comparison with TAU was 46.7% (95% CI = 31.4 to 61.9), with NNT = 3 (95% CI = 1.6–3.2), meaning that 3 patients would need to be treated with TAU + NAT-FM for one of them to become a responder, which would otherwise have not been possible in the TAU group.

The responder group showed significantly higher scores with a small effect size (Cohen’s *d* ≤ 0.49) in BMI (non-responders: 26.22 ± 5.91; responders: 28.25 ± 5.31), age (non-responders: 51.11 ± 9.31; responders: 55.87 ± 6.81), years of illness (non-responders: 16.30 ± 8.98; responders: 21.66 ± 12.49), and in perspective taking (CERQ) (non-responders: 4.91 ± 2.05; responders: 5.94 ± 2.34). The non-responder group reported significantly higher scores with a small effect size (Cohen’s *d* ≤ 0.43) in anxiety (non-responders: 14.16 ± 3.88; responders: 12.45 ± 4.06), depression (non-responders: 12.38 ± 4.21; responders: 10.66 ± 4.36), and pain catastrophizing thoughts (non-responders: 30.54 ± 11.34; responders: 25.50 ± 12.66). There were no significant differences between groups in terms of any other sociodemographic or clinical variables.

### 3.6. Effects of the Different Activities Included in TAU + NAT-FM Treatment: Intra-Session Assessments (EMA)

**Figure 2:**
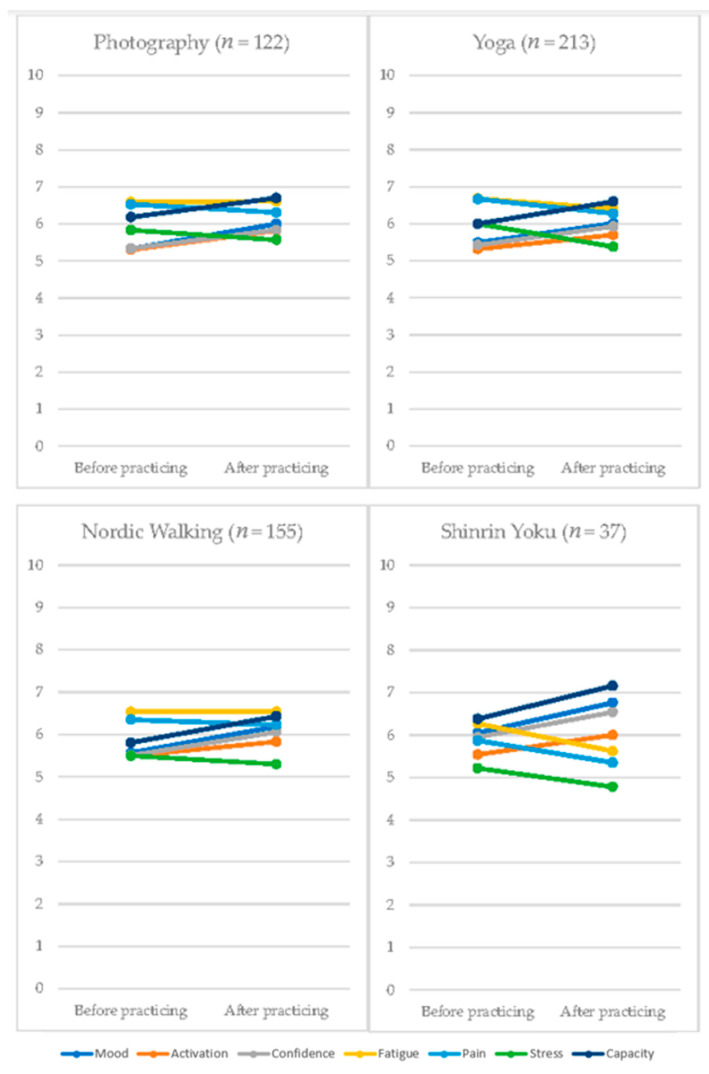
Effect of the different activities included in NAT-FM treatment: intra-session assessments (EMA). Note: all *p* values < 0.05 except for fatigue in photography (*p* = 0.92), and fatigue (*p* = 0.95) and pain (*p* = 0.15) in Nordic walking. NAT-FM, nature activity therapy for fibromyalgia.

To explore the effect of the different activities included in the TAU + NAT-FM treatment (i.e., yoga, Nordic walking, nature photography, and Shinrin Yoku), we analyzed, for each activity, the change produced in affective valence, arousal, dominance, fatigue, pain, stress, and self-efficacy after practicing the activity. The results are displayed in Figure 2.

After practicing Nordic walking, the patients reported an improvement in affective valence, arousal, dominance, stress and self-efficacy (all *p* values < 0.05), except for pain and fatigue. After yoga, the patients showed an improvement in all variables analyzed (all *p* values < 0.05). Photography was associated with improvements in all variables (all *p* values < 0.05), except fatigue. And Shinrin Yoku, like yoga, produced an improvement in all variables analyzed.

### 3.7. The Role of Kinesiophobia, Pain Catastrophism, Perceived Competence, and Cognitive Emotion Regulation Strategies as Treatment Mediators

**Figure 3:**
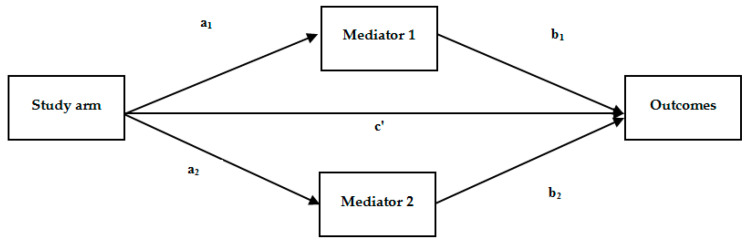
Multiple mediational model with two mediators (generic example). When the relationship between study arm and outcome is explained by the mediators (Mediator 1 and Mediator 2), it is called full mediational model. Furthermore, the model is consistent with partial mediation when the study arm still has a direct effect on outcome after including the mediators.

We computed bivariate correlational analyses between pre–posttreatment differences in the primary and secondary outcomes and baseline to 6-week changes in the process variables within the TAU + NAT-FM group (Supplementary Table S1) following the model drawn in the Figure 3. The path analysis results are detailed in Table 6 and illustrated in Figure 2.

Two of the 10 tested path models did not show a partial or total mediation effect. Some of the 8 models with significant mediational effects (i.e., Fatigue, HADS-D, PANAS-NA, and RSES) did not yield significant direct paths between the treatment arm and clinical outcomes, meaning that there was a total mediation; in the remaining cases, the mediation was partial. 

In the mediational model for the FIQR, the two significant mediators were TSK and PPCS (partial mediation), and the same was observed for HADS-A, HADS-D, and SF-36. In the case of VAS Pain, the treatment arm predicted the change in TSK and in PPCS at 6-week assessment, which in turn predicted the change in VAS pain scores at posttreatment (partial mediation). For VAS Fatigue, the treatment arm predicted the change in TSK, which in turn predicted the posttreatment change in this clinical outcome (total mediation). The mediational model for PANAS-NA resulted in only one mediator (PPCS), which was also the case for RSES (CERQ-positive reappraisal). No significant mediators were found for PSS and PANAS-PA.

## 4. Discussion

To our knowledge, this is the first study to report the effectiveness of a multicomponent treatment that includes the combination of PNE, TE, CBT, MT and exposure to nature for the treatment of FM. A recent RCT; the FIBROWALK study [27], has demonstrated the effectiveness of combining these components, but it did not integrate exposure to nature.

The primary aim of the present study was to analyze the effectiveness of a 12-week multicomponent treatment based on PNE, TE, NA, CBT, MT, and nature exposure, as an add-on to TAU to improve a wide range of FM-related outcomes. Data analyses revealed that TAU + NAT-FM (added to TAU) compared with TAU alone was an effective adjuvant therapy for patients with FM. TAU + NAT-FM achieved significant results with a large effect size at posttreatment on the primary outcome (i.e., functional impairment) and on the following secondary variables: pain, fatigue, anxiety, depression and physical function; and on the process variables of kinesiophobia, pain catastrophizing thoughts, personal perceived competence, cognitive emotion regulation subscales (refocusing, planning, positive reappraisal and catastrophizing). These large effect sizes had already been achieved at 6-weeks of treatment regarding functional impairment, anxiety, kinesiophobia, personal perceived competence, and positive reappraisal. In addition, significant results with moderate effect size were obtained at six-weeks for pain, fatigue, physical function, personal perceived competence, refocusing (CERQ), and planning (CERQ). Our results indicate that these beneficial outcomes are achieved as therapy progresses, but at six-weeks there is already a significant (but not yet complete) improvement in the main outcomes analyzed in these FM patients. Further studies should be conducted to investigate how extending the therapy to include more sessions could have an impact on (1) the therapeutic effect achieved and (2) maintenance of the effects achieved post-therapy.

In accordance with the findings reported in the previous literature [16,17,27] the TAU + NAT-FM intervention appears to be an effective multicomponent intervention for improving FM symptoms, at least in terms of functional impairment, pain, fatigue, anxiety, depression, physical function, and kinesiophobia. More recently [24], education, psychological support and exercise therapy have been shown to be the most effective combined strategies. To date, studies have reported effect sizes of small to moderate magnitude. Although there are yet no curative treatments for FM, when using the TAU + NAT-FM intervention, the active group not only showed that this intervention had a large effect on the main outcomes, but 66.7% achieved a decrease in their FIQR score by at least 20% when compared with baseline assessment, 1.3% achieved a decrease of at least 70%, 8.1% achieved a decrease of at least 60%, along with an NNT of 3. The responder group had higher BMI, age, years of illness and perspective, and lower baseline scores on anxiety, depression, and pain catastrophizing thoughts. The non-responder group, as shown in the TAU + NAT-FM and FIBROWALK intervention, had, at least, higher scores on depression according to previous literature [27,69,70]. 

The FIBROWALK study [27] also found significant differences with large effect size at posttreatment on the primary outcome: functional impairment, and on pain, physical function and kinesiophobia (secondary outcomes), along with significant differences of moderate effect size at posttreatment for fatigue, anxiety and depression (secondary outcomes. Further, 51.85% reached the criterion of ≥20% FIQR reduction, a total of 7 patients (5.2%) showed a reduction in their FIQR score by more than 70%, with an NNT of 2. More studies are needed to confirm the other significant differences that have been reported with the TAU + NAT-FM intervention.

The improvements generated by the TAU + NAT-FM intervention on the effect sizes of certain outcomes could be due not only to the effect of combining these interventions but also to the exposure to nature. Our preliminarily assessment of the effect of the various activities included in the TAU + NAT-FM intervention has revealed that these activities could be useful for improving certain emotional and cognitive targets. Yoga and Shinrin Yoku appear to be promising for the improvement of affective valence, arousal, dominance, fatigue, pain, stress and self-efficacy; nature photography appears to improve all variables except for fatigue; whilst Nordic walking could improve all variables apart from fatigue and pain. The results of EMA intrasession provided preliminary evidence of the positive effect of the psychical activities practiced in nature. Consistent with previous research, interventions based on physical activity in nature yielded a positive effect on emotional, cognitive and behavioral functioning [29,30,31,32,33,34,37].

Future studies on this type of multicomponent intervention should identify the elements that make the most significant contribution to the effects of the TAU + NAT-FM, and should also focus on long-term clinical outcomes compared with TAU, whilst providing more detailed information about the potential role played by the nature-based activities explored in this study.

Mediation analyses revealed that pre-post changes in kinesiophobia and personal perceived competence partially mediated the relationships between study condition and functional impact, anxiety, depression and physical function. A total mediation effect was found for kinesiophobia on the relationship between study condition and fatigue, for perceived competence in the link between study condition and negative affect, and for positive reappraisal (CERQ) in the study condition and self-esteem (RSES) relationship. Our results suggest that these mediators could explain a large part of the improvements, and we should focus our attention on these mediators for improving the TAU + NAT-FM therapeutic process. However, in this regard, it would be of interest for future research to evaluate whether other mediators could be involved in the main components of TAU + NAT-FM.

### Limitations and Strengths of This Study

Due to the fact that this clinical research was conducted by the main researcher (M.S.) in the context of real-life clinical practice in a specialized unit of a tertiary referral hospital, and that it is currently not possible to change these conditions, the main limitations of this work are the same as those described in a previous study [27]. To sum up, these limitations were: (a) the impossibility of applying strict selection criteria, (b) no follow up of the sample beyond post treatment, (c) absence of blinding in the group assignment, (d) the intervention was conducted by only one therapist, although the therapist is also a physiotherapist, psychologist and mountain sports technician, which provides the knowledge required to apply this multicomponent therapy; (e) the high number of patients per group (20), and (f) the high chronic disease duration with high baseline values of the main core symptoms of FM of the patients recruited. Consideration of the relevance of this approach as a complementary model of health intervention and its evaluation in primary care patients could help to resolve the limitations described above.

With regard to the effect of the various activities included in TAU + NAT-FM, certain limitations are worth noting. First, not every activity was performed with the same frequency, with yoga being the one that was most often practiced and registered. The format of the sessions and the subsequent assessment of the effect of the activity (i.e., one activity began immediately after the other had finished) means that it is not possible to reliably compare the activities, as those which were performed second would quite possibly be affected by the previous activity. For these reasons, the results presented must be regarded as preliminary and, in order to assess the differential effect of each activity, further studies could try to adapt this methodology, assessing only the effect of the first activity in each session, and ensuring that a similar number of records is obtained for each activity.

Due to the lack of follow-up, the mediation analyses could only be performed with the changes observed in the process variables between the baseline evaluation and the six-week assessment. These results should be replicated considering, if possible, how the pre-post treatment changes in these variables could mediate the long-term changes in the main variables (i.e., pain, fatigue, and functional impact, among others). Moreover, some other relevant process variables could be included in further studies, such as, for instance, psychological inflexibility or the mindfulness facet “acting with awareness”, which have proved to be significant mediators of long-term changes produced by a mindfulness-based intervention for patients with FM [15].

To our knowledge, this is also the first study to demonstrate the effectiveness of a multicomponent treatment that specifically integrates nature exposure in patients with FM. There are a number of studies supporting the effectiveness of each or a combination of two or more components of the intervention that constitute this multicomponent approach [19,20,21,22,23,24,25,26,29,30,31,32,33,34,36]. Although integrating the different therapeutic interventions was a complex process, the TAU + NAT-FM approach was designed with a clear and replicable methodology [27,28,36] and it is based on an empirically validated framework. The relatively low dropout rate was made possible due to the use of certain therapeutic adherence strategies whereby phone and mail contacts were established with FM patients [27].

## 5. Conclusions

In conclusion, the TAU + NAT-FM treatment is presented as the first intervention that integrates PNE, TE, CBT, MT, and exposure to the natural environment. It is based on recognized scientific evidence reported in various studies in the three central components of treatment (NAT: nature activity therapy). The results of this study suggest that the TAU + NAT-FM intervention (added to TAU) could emerge as an add-on therapy conceived as a new generation of therapeutic intervention that not only improves the core symptoms of this prevalent and costly disease in comparison with usual treatments, but also one that is of benefit both socially, due to the high use of public resources, and economically, due to the high consumption of health resources and job losses resulting from this illness. It also provides novel and useful information to promote a future paradigm shift in the management of FM, chronic pain in general, and various other health problems by combining different approaches in natural contexts.

This study highlights the need to consider the relevance of this approach as a complementary model of health intervention that could be applied and evaluated in primary care patients.

## Figures and Tables

**Table 1:** Outline of active group sessions in NAT-FM treatment.

**Review Phase (15 min)**
• Comment on the duties of the previous session.
• Review of contents of the previous session.
**Main Phase (1 h 40 min)**
• 20 min Pain Neuroscience Education (PNE).
• 40 min Exercise therapy (TE) or Nature Activity (NA) or both (20 min each).
• 20 min Cognitive Behavioral Therapy (CBT).
• 20 min Mindfulness training (MT)
**Sessions:**
1. PNE (1,2) + CBT (1) + MT (1)
2. PNE (3,4) + TE (2) + CBT (2, 13–18) + MT (2)
3. PNE (5,6) + TE (2) + CBT (3, 19–23) + MT (3) + NA (3)
4. PNE (7,8) + TE (4) + CBT (4, 24–28) + MT (4)
5. PNE (9,10) + TE (4) + CBT (5, 15–28) + MT (5) + NA (3)
6. PNE (11) + TE (2) + CBT (6, 29–34) + MT (6) + TE (4)
7. PNE (12) + TE (4) + CBT (7,8, 35–40) + MT (7,8) + NA (5)
8. PNE (13) + TE (4) + CBT (9, 41–44) + MT (9) + NA (3)
9. PNE (14) + TE (2) + CBT (10, 45–48) + MT (10)
10. Family Session (PNE 1–16) + NA (3)
11. PNE (15) + TE (4) + CBT (11, 47–48) + MT (11) + NA (5)
12. PNE (16) + TE (4) + CBT (12, 18, 28, 34, 40, 48) + MT (12)
**Homework (5 min)**
• TE (1): first month once per week, second month twice per week, and third month three times per week. • Cognitive (related to CBT and MT) and physical tasks to do at home to increase the patient’s resistance involving a constant challenge for them.

Note: The numbers in parentheses from 1 to 16 of pain neuroscience education (PNE), from 1 to 48 of CBT, from 1 to 5 of TE/NA and from 1 to 12 of mindfulness training (MT) are explained on Table 2. The exercise therapy was designed following the recommendations of the American College of Sports Medicine (ACSM) and was performed following the same procedures described elsewhere (Serrat 2020). TE/NA, therapeutic exercise with nature exposition.

**Table 2:** Steps of the multicomponent treatment: PNE, exercise therapy, cognitive behavioral therapy (CBT), mindfulness and nature exposure.

**Pain Neuroscience Education (PNE)**
Disassembling beliefs. Danger signals: modulation and modification. Concept of pain, fatigue, and pain system. Concept of central nervous system and central sensitization. The role of the brain. Acute vs. Chronic Pain: The purpose of acute pain and how it originates in the nervous central system (CNS). Pain vs. damage. Pain neuromatrix theory and representation of the virtual body. Nociception, nociceptors, action potential, peripheral sensitization, and synapses. Ascending and descending inhibitory pathways, spinal cord. Relationship with attention, perception, pain cognitions, and pain behaviors. Allodynia and hyperalgesia, hypersensitivity of nervous central system. Pain memory, pain perception, and autoimmune evaluation error. Relationship with stress. Etiology. Neuroplasticity and how the pain becomes chronic. Relationship with emotions. Re-education, gradual activity, and exercise therapy.
**Exercise Therapy (TE)/Nature Activities (NA) Used to Work the Cognitive Targets**
TE: Hiking * TE: Yoga ** NA: Photography TE: Nordic Walking ** NA: Shinrin Yoku ***
**Cognitive Behavioral Therapy (CBT)**
**General Issues:** Relaxation and breathing. Modulating factors of pain. Catastrophizing and fear of movement. Painful experiences: confrontation. Vital values and setting goals. Organization of time. Sleep patterns. Sexual issues. Handling of attention. Cognitive restructuring. Emotional regulation and assertiveness. Troubleshooting. **Cognitive Targets:** **Positive affect** 13. Understand the concept of positive emotions. 14. Recognize the relationship between positive emotions, pain, and fatigue. 15. Recognize the importance of actively searching for sources of positive emotions. 16. Identify sources of positive emotions in the context of the sessions. 17. Learn to pay attention to stimuli/conditions that generate positive emotions. 18. Transfer: How to work positive affect in day life. **Self-efficacy** 19. Understand the concept of self-efficacy. 20. Recognize the relationship between self-efficacy, pain, and fatigue. 21. Recognize the importance of adapting self-efficacy to real capacity. 22. Become aware of the process of self-efficacy elaboration. 23. Recognize the dynamics of self-efficacy. 24. Identify the sources of self-efficacy. 25. Identify the biases in the creation of self-efficacy. 26. Learn how to stop and replace the biases in the creation of self-efficacy. 27. Relationship between self-efficacy and self-esteem. 28. Transfer: How to work self-efficacy in day life. **Negative affect** 29. Understand the concept of negative emotions. 30. Recognize the relationship between negative emotions, pain, and fatigue. 31. Recognize the adaptive function of negative emotions 32. Assess the importance of emotional regulation to reduce negative emotions 33. Learn to pay attention to the stimuli/conditions that generate negative emotions 34. Transfer: How to reduce negative affect to everyday life. **Catastrophic thinking** 35. Understand the concept of catastrophism. 36. Recognize the relationship between catastrophism, pain, and fatigue. 37. Learn to recognize the catastrophic thoughts. 38. Learn to pay attention to the catastrophic thoughts 39. Learn to stop and replace the catastrophic thoughts. 40. Transfer: How to work catastrophism on an everyday basis. **Emotional regulation** 41. Understand the concept of emotional regulation. 42. Recognize the relationship between emotional regulation, pain, and fatigue. 43. Identify the 9 types of cognitive regulation of emotions. 44. Identify the relationship between emotional regulation, pain, and fatigue. 45. Learn to pay attention to emotional regulation. 46. Learn to identify the type of emotional regulation usually employed in day life. 47. Learn to stop and subtract inappropriate emotional regulation for a proper one. 48. Transfer: How to work emotional regulation in everyday life.
**Mindfulness Training (MT)**
An Introduction to Body Scanning. Elementary Awareness. Sitting Practice and introduction to Yoga. Mindfulness and the Brain. Mindfulness and communication: guilt, empathy, and conflict management. Responding vs. reacting. Dig deeper into personal practice. Mindfulness and Compassion: Strength vs. Cooperation. Stress Management. Thoughts Management. Management of difficult emotions or feelings. Dig deeper into personal practice.

Note: * Hiking is a homework assignment to do as an exercise therapy with cognitive targets; ** hiking, yoga and Nordic walking has been done as an exercise therapy following the same procedures described elsewhere [27]; *** Shinrin Yoku is understood as mindfulness in a natural context.

**Table 3:** Demographic characteristics of treatment as usual (TAU) + NAT-FM and TAU participants.

	TAU + NAT-FM (*n* = 84)	TAU (*n* = 85)	t/*χ*^2^	*p*
**General measures**				
Gender, n of women (%)	82 (97.60)	85 (100)	0.74	0.39
Age, M (SD)	54.12 (8.62)	53.15 (9.06)	0.32	0.57
BMI (kg/m^2^), M (SD)	27.65 (5.49)	26.75 (5.75)	0.48	0.62
Years of illness, M (SD)	19.61 (11.99)	16.75 (9.74)	2.92	0.08
Married or in couple, n (%)	57 (67.8)	43 (50.6)	10.41	0.34
Cohabitating, n (%)	38 (44.2)	69 (81.2)	1.84	0.39
Secondary studies, n (%)	38 (45.3)	46 (54.7)	7.45	0.15
Labor assets, n (%)	43 (52.0)	40 (48.0)	18.52	0.09
Accreditation of disability in process, n (%)	30 (36.0)	54 (64.0)	1.13	0.29
**Comorbidity with CSS, f (%)**				
Chronic fatigue	71 (84.6)	72 (85.3)	0.32	0.59
Multiple chemical sensitivity	30 (35.8)	25 (29)	1.72	0.17
Irritable bowel syndrome	38 (45.9)	39 (46.3)	0.08	0.70
Migraines	50 (59)	49 (57.8)	0.07	0.92
**Medication, f (%)**				
Mix of more than two medications	27 (32.9)	41 (47.7)	4.71	0.41

Note: The values represent means (M) and standard deviation (SD) or frequency (n) and percentages (%), in their respective order of presentation. BMI = body mass index. Significant values (*p* < 0.05) should be shown in bold. There are no statistically significant group differences. CSS, central sensitization syndromes.

**Table 4:** Descriptive statistics and between-group analyses for primary and secondary outcomes (intention to treat (ITT) approach).

	TAU M (SD)	TAU + NAT-FM M (SD)	TAU vs. NAT-FM		
			d	t (*p*)	B (95% CI)
**Primary outcome**					
FIQR (0–100) *					
Baseline	73.21 (14.72)	73.07 (13.79)			
6 weeks	69.68 (13.36)	58.78 (18.70)	1.13	−5.29 (<0.001)	−10.96 (−15.04 to −6.88)
Post−treatment	69.18 (17.88)	50.69 (18.05)	1.83	−8.16 (<0.001)	−18.07 (−22.43 to −13.72)
**Secondary outcomes**					
VAS PAIN (0–10) *					
Baseline	7.80 (1.61)	7.74 (1.52)			
6 weeks	7.52 (1.59)	6.78 (1.99)	0.66	−3.02 (0.003)	−0.77 (−1.28 to −0.27)
Post-treatment	7.47 (1.79)	5.60 (1.98)	5.62	−6.53 (<0.001)	−1.79 (−2.33 to −1.25)
VAS FATIGUE (0–10) *					
Baseline	7.76 (1.91)	7.61 (1.89)			
6 weeks	7.32 (2.09)	5.98 (2.10)	0.77	−3.10 (0.002)	−1.26 (−2.06 to −0.46)
Post-treatment	7.08 (2.34)	5.58 (2.00)	0.93	−3.87 (<0.001)	−1.53 (−2.31 to −0.75)
HADS-A (0–21) *					
Baseline	13.13 (4.22)	13.95 (3.80)			
6 weeks	12.35 (4.07)	11.03 (4.25)	0.99	−4.52 (<0.001)	−2.20 (−3.16 to −1.24)
Post-treatment	12.68 (4.63)	10.16 (4.19)	1.59	−7.08 (<0.001)	−3.51 (−4.48 to −2.53)
HADS-D (0–21) *					
Baseline	11.49 (4.64)	11.27 (3.71)			
6 weeks	11.22 (5.02)	9.66 (4.47)	0.49	−2.23 (0.027)	−1.10 (−2.07 to −0.13)
Post-treatment	11.67 (5.18)	8.18 (4.42)	1.45	−6.40 (<0.001)	−3.37 (−4.41 to −2.34)
SF-36 (0–100) *					
Baseline	26.04 (18.11)	27.03 (18.85)			
6 weeks	28.24 (17.38)	35.09 (20.47)	0.53	2.41 (0.017)	5.51 (1.00 to 10.02)
Post-treatment	25.07 (15.86)	43.42 (20.92)	1.59	7.01 (<0.001)	17.15 (12.34 to 21.96)
PANAS-PA (0–50) *					
Baseline	12.26 (4.38)	11.95 (5.79)			
6 weeks	12.20 (4.30)	12.81 (5.39)	0.19	1.00 (0.319)	0.79 (−0.77 to 2.35)
Post-treatment	13.01 (4.03)	14.11 (4.28)	0.40	2.07 (0.039)	1.68 (0.08 to 3.27)
PANAS-NA (0–50) *					
Baseline	14.34 (5.81)	13.84 (6.08)			
6 weeks	13.94 (5.13)	13.22 (4.83)	0.13	−0.37 (0.714)	−0.29 (−1.87 to 1.28)
Post-treatment	14.95 (4.50)	13.12 (4.24)	0.28	−1.38 (0.167)	−1.14 (−2.75 to 0.48)
RSES (10–40) *					
Baseline	15.41 (3.57)	16.03 (3.36)			
6 weeks	15.48 (2.57)	16.60 (2.70)	0.37	1.23 (0.219)	0.68 (−0.40 to 1.75)
Post-treatment	16.25 (3.45)	16.53 (2.25)	0.03	−0.24 (0.809)	−0.13 (−1.20 to 0.94)
PSS (0–16) *					
Baseline	8.88 (2.15)	8.93 (2.31)			
6 weeks	8.81 (1.90)	7.91 (1.87)	0.43	−1.88 (0.062)	−0.77 (−1.57 to 0.04)
Post-treatment	8.88 (2.20)	8 (1.87)	0.37	−1.66 (0.098)	−0.67 (−1.47 to 0.12)

Note: Mean and SD are not adjusted. When the Benjamini–Hochberg correction was applied to correct for multiple comparisons, the following effects were not statistically significant: positive affect and negative affect schedule (PANAS) positive (*p* = 0.059). The number of participants varied across assessment periods due to dropouts (see flow chart). Significant values (*p* < 0.05) are shown in bold. * The baseline level of the variable is a significant covariate in the model. B, regression coefficients; CI, confidence interval; d, Cohen’s d as an effect size measure; ITT, intention-to-treat; NAT-FM; TAU, treatment-as-usual.

**Table 5:** Descriptive statistics and between-group analyses for process variables (ITT approach).

	TAU M (SD)	TAU+NAT-FM M (SD)	TAU vs. TAU+NAT-FM		
			d	t (*p*)	B (95% CI)
TSK (11–44) *					
Baseline	29.92 (7.58)	29.23 (7.40)			
6 weeks	25.59 (6.46)	21.36 (6.83)	1.18	**−5.35 (<0.001)**	−4.73 (−6.47 to −2.98)
Post-treatment	28 (7.44)	17.95 (4.97)	2.2	**−9.65 (<0.001)**	−9.15 (−11.01 to −7.28)
PCS (0–52) *					
Baseline	27.72 (12.65)	27.04 (11.33)			
6 weeks	26.72 (13.25)	17.83 (9.56)	1.21	**−5.53 (<0.001)**	−7.30 (−9.90 to −4.70)
Post-treatment	27.49 (13.35)	13.53 (8.87)	2.03	**−8.93 (<0.001)**	−12.56 (−15.33 to −9.80)
PPCS					
Baseline	25.05 (7.84)	23.77 (7.98)			
6 weeks	25.35 (8.22)	27.50 (8.08)	0.72	**3.44 (0.001)**	3.73 (1.59 to 5.86)
Post-treatment	24.57 (8.50)	28.67 (8.62)	1.2	**5.47 (<0.001)**	6.17 (3.95 to 8.38)
CERQ Acceptance (0–20) *					
Baseline	6.46 (2.33)	6.19 (2.27)			
6 weeks	6.28 (2.37)	7.02 (2.12)	0.47	**2.28 (0.023)**	0.93 (0.13 to 1.73)
Post-treatment	6.77 (2.28)	7.47 (2.15)	0.53	**2.52 (0.012)**	1.04 (0.23 to 1.84)
CERQ Self-blame (0–20) *					
Baseline	5.14 (2.39)	4.47 (2.26)			
6 weeks	4.63 (2.01)	4.24 (2.09)	0.12	1.14 (0.256)	0.40 (−0.29 to 1.08)
Post-treatment	4.43 (2.13)	3.74 (1.96)	0.14	0.03 (0.980)	0.01 (−0.68 to 0.70)
CERQ Rumination (0–20) *					
Baseline	6.45 (2.33)	5.89 (2.11)			
6 weeks	5.80 (2.21)	5.36 (2.06)	0.03	0.30 (0.763)	0.11 (−0.60 to 0.82)
Post-treatment	5.84 (2.48)	4.70 (2.10)	0.47	−1.60 (0.110)	−0.59 (−1.32 to 0.13)
CERQ Refocusing (0–20) *					
Baseline	4.48 (1.94)	4.26 (1.92)			
6 weeks	4.70 (1.99)	5.19 (2.11)	0.52	**2.50 (0.013)**	0.80 (0.17 to 1.42)
Post-treatment	4.77 (2.13)	5.82 (2.20)	0.99	**4.49 (<0.001)**	1.47 (0.83 to 2.11)
CERQ Planning (0–20) *					
Baseline	5.58 (2.11)	5.41 (2.20)			
6 weeks	5.63 (2.17)	6.38 (2)	0.58	**2.71 (0.007)**	0.98 (0.27 to 1.70)
Post-treatment	5.28 (2.23)	6.40 (2.11)	0.83	**3.76 (<0.001)**	1.38 (0.65 to 2.11)
CERQ Positive reappraisal (0–20) *					
Baseline	5.12 (2.11)	4.96 (2.42)			
6 weeks	4.80 (1.77)	6.07 (2.26)	0.92	**4.17 (<0.001)**	1.44 (0.76 to 2.11)
Post-treatment	4.67 (2.23)	6.42 (2.28)	1.42	**6.27 (<0.001)**	2.19 (1.50 to 2.87)
CERQ Perspective (0–20) *					
Baseline	5.45 (2.25)	5.45 (2.23)			
6 weeks	5.52 (2.08)	6.03 (2.14)	0.17	0.79 (0.431)	0.27 (−0.40 to 0.93)
Post-treatment	5.23 (1.95)	6.46 (2.51)	0.71	**3.15 (0.002)**	1.12 (0.42 to 1.81)
CERQ Catastrophizing (0–20) *					
Baseline	5.34 (2.37)	5.27 (2.17)			
6 weeks	4.87 (2.07)	4.09 (1.83)	0.47	**−2.04 (0.043)**	−0.65 (−1.27 to −0.02)
Post-treatment	5.04 (2.21)	3.70 (1.72)	0.85	**−3.64 (<0.001)**	−1.18 (−1.82 to −0.54)
CERQ Blame others (0–20) *					
Baseline	3.45 (2.16)	3.42 (2.20)			
6 weeks	3.20 (1.65)	2.97 (1.62)	0.14	−1.23 (0.219)	−0.34 (−0.88 to 0.20)
Post-treatment	3.20 (1.70)	3.02 (1.72)	0.11	−0.50 (0.620)	−0.14 (−0.69 to 0.41)

Note: Mean and SD are not adjusted. When the Benjamini–Hochberg correction was applied to correct for multiple comparisons, the following effects were not statistically significant: CERQ catastrophizing (*p* = 0.063). The number of participants varied across assessment periods due to dropouts (see flow chart). Significant values (*p* < 0.05) are shown in bold. * The baseline level of the variable is a significant covariate in the model. B, regression coefficients; CI, confidence interval; d, Cohen’s d as an effect size measure; ITT, intention-to-treat; NAT-FM; TAU, treatment-as-usual; TSK, Tampa scale for kinesiophobia; PCS, pain catastrophizing scale; PPCS personal perceived competence scale; CERQ, cognitive emotion regulation questionnaire.

**Table 6:** Direct and bootstrap indirect effects in the multiple mediational models of TAU+NAT-FM vs. TAU (effects of pre- to mid-treatment changes in process variables on pre-to-post changes in primary and secondary outcomes).

Outcome and Mediators (R^2^)	Direct Effects				Indirect Effects			
	Path	Coeff.	SE	*p*	Path	Boot.	SE	95% CI
FIQR (0.48)								
M1 = TSK (0.17)	a_1_	−5.39	1.21	<0.001	a_1_ × b_1_	−4.10	1.29	−7.22 to −2.06
	a_2_	7.50	1.46	<0.001				
M2 = PPCS (0.21)	b_1_	0.76	0.19	<0.001	a_2_ × b_2_	−4.23	1.83	−8.39 to −1.25
	b_2_	−0.56	0.19	0.002				
	c’	−9.76	2.79	<0.001				
VAS Pain (0.30)								
M1 = TSK (0.17)	a_1_	−5.39	1.19	<0.001	a_1_ × b_1_	−0.44	0.19	−0.89 to −0.13
	a_2_	7.50	1.46	<0.001				
M2 = PPCS (0.21)	b_1_	0.08	0.03	0.008	a_2_ × b_2_	−0.38	0.19	−0.82 to −0.07
	b_2_	−0.05	0.02	0.010				
	c’	−1.00	0.39	0.010				
VAS Fatigue (0.20)								
M1 = TSK (0.17)	a_1_	−5.39	1.18	<0.001	a_1_ × b_1_	−0.64	0.22	−1.16 to −0.29
	b_1_	0.12	0.04	0.004				
	c’	−0.76	0.49	0.120				
HADS Anxiety (0.41)								
M1 = TSK (0.17)	a_1_	−5.39	1.19	<0.001	a_1_ × b_1_	−0.56	0.29	−1.28 to −0.11
	a_2_	7.50	1.46	<0.001				
M2 = PPCS (0.21)	b_1_	0.10	0.05	0.022	a_2_ × b_2_	−1.31	0.50	−2.54 to −0.54
	b_2_	−0.17	0.05	<0.001				
	c’	−1.39	0.58	0.017				
HADS Depression (0.37)								
M1 = TSK (0.17)	a_1_	−5.39	1.21	<0.001	a_1_ × b_1_	−0.70	0.29	−1.40 to −0.23
	a_2_	5.40	1.45	<0.001				
M2 = PPCS (0.21)	b_1_	0.13	0.05	0.008	a_2_ × b_2_	−1.17	0.57	−2.47 to −0.28
	b_2_	−0.16	0.06	0.011				
	c’	−1.60	0.83	0.055				
SF36 (0.40)								
M1 = TSK (0.17)	a_1_	−5.39	1.19	<0.001	a_1_ × b_1_	3.06	1.63	0.48 to 7.09
	a_2_	7.50	1.46	<0.001				
M2 = PPCS (0.21)	b_1_	−0.57	0.27	0.036	a_2_ × b_2_	4.47	1.84	1.59 to 8.92
	b_2_	0.60	0.20	0.003				
	c’	10.57	3.07	0.001				
PANAS—(0.12)								
M1 = PPCS (0.21)	a_1_	7.50	1.47	<0.001	a_1_ × b_1_	−1.43	0.53	−2.60 to −0.49
	b_1_	−0.19	0.06	0.003				
	c’	0.98	1.06	0.355				
RSES (0.06)								
M1 = CERQ Positive Reappraisal (0.17)	a_1_	1.91	0.41	<0.001	a_1_ × b_1_	−0.66	0.34	−1.45 to −0.09
	b_1_	−0.35	0.17	0.040				
	c’	0.41	0.67	0.539				

Note: For clarity, a generic example of a multiple mediational model (with two mediators) is displayed in Figure 2. CERQ, cognitive emotion regulation questionnaire; HADS, hospital anxiety and depression scale; FIQR, fibromyalgia impact questionnaire—revised; PANAS, positive affect negative affect scale; PPCS, personal perceived competence scale; RSES, Rosenberg self-esteem scale; SF-36, 36 items short form survey; TSK, Tampa scale for kinesiophobia; VAS, visual analogue scale.

## Supplementary Materials

The following are available online at https://www.mdpi.com/2077-0383/9/10/3348/s1, Table S1: correlation table between pre-six week changes in process variables (TSK, PCS, PPCS and CERQ) and pre-post changes in main variables.

## References

[1] Gayà T.F., Ferrer C.B., Mas A.J., Seoane-Mato D., Reyes F.Á., Sánchez M.D., Dubois C.M., Sánchez-Fernández A.S., Vargas L.M.R., Morales P.V.G. (2020). Prevalence of fibromyalgia and associated factors in Spain. Proyecto EPISER2016. Clin. Exp. Rheumatol.

[2] Gormsen L., Rosenberg R., Bach F.W., Jensen T.S. (2010). Depression, anxiety, health-related quality of life and pain in patients with chronic fibromyalgia and neuropathic pain. Eur. J. Pain.

[3] Häuser W., Ablin J., Fitzcharles M.-A., Littlejohn G., Luciano J.V., Usui C., Walitt B. (2015). Fibromyalgia. Nat. Rev. Dis. Prim..

[4] Wolfe F., Clauw D.J., Fitzcharles M.-A., Goldenberg D.L., Häuser W., Katz R.L., Mease P.J., Russell A.S., Russell I.J., Walitt B. (2016). 2016 Revisions to the 2010/2011 fibromyalgia diagnostic criteria. Semin. Arthritis Rheum..

[5] Wolfe F., Walitt B., Perrot S., Rasker J.J., Häuser W. (2018). Fibromyalgia diagnosis and biased assessment: Sex, prevalence and bias. PLoS ONE.

[6] Schaefer C., Chandran A., Hufstader M., Baik R., McNett M., Goldenberg D., Gerwin R., Zlateva G. (2011). The comparative burden of mild, moderate and severe Fibromyalgia: Results from a cross-sectional survey in the United States. Health Qual. Life Outcomes.

[7] Staud R. (2008). The Role of Peripheral Input for Chronic Pain Syndromes like Fibromyalgia Syndrome. J. Musculoskelet. Pain.

[8] Woolf C.J. (2011). Central sensitization: Implications for the diagnosis and treatment of pain. Pain.

[9] Harte S.E., Harris R.E., Clauw D.J. (2018). The neurobiology of central sensitization. J. Appl. Biobehav. Res..

[10] Nijs J., Van Houdenhove B., Oostendorp R.A. (2010). Recognition of central sensitization in patients with musculoskeletal pain: Application of pain neurophysiology in manual therapy practice. Man. Ther..

[11] Wolfe F., Clauw D.J., Fitzcharles M.-A., Goldenberg D.L., Katz R.S., Mease P., Russell A.S., Russell I.J., Winfield J.B., Yunus M.B. (2010). The American College of Rheumatology Preliminary Diagnostic Criteria for Fibromyalgia and Measurement of Symptom Severity. Arthritis Care Res. (Hoboken).

[12] Feliu-Soler A., Borràs X., Peñarrubia-María M., Rozadilla-Sacanell A., D’Amico F., Moss-Morris R., Howard M., Fayed N., Soriano-Mas C., Puebla-Guedea M. (2016). Cost-utility and biological underpinnings of Mindfulness-Based Stress Reduction (MBSR) versus a psychoeducational programme (FibroQoL) for fibromyalgia: A 12-month randomised controlled trial (EUDAIMON study). BMC Complement. Altern. Med..

[13] Haugmark T., Hagen K.B., Provan S.A., Bærheim E., Zangi H.A. (2018). Effects of a community-based multicomponent rehabilitation programme for patients with fibromyalgia: Protocol for a randomised controlled trial. BMJ Open.

[14] Häuser W., Ablin J., Perrot S., Fitzcharles M.-A. (2017). Management of fibromyalgia: Practical guides from recent evidence-based guidelines. Pol. Arch. Intern. Med..

[15] Pérez-Aranda A., Feliu-Soler A., Montero-Marín J., García-Campayo J., Andrés-Rodríguez L., Borràs X., Rozadilla-Sacanell M., Peñarrubia-Mari T., Angarita-Osorio N., McCracken L.M. (2019). A randomized controlled efficacy trial of Mindfulness-Based Stress Reduction compared to an active control group and usual care for fibromyalgia: The EUDAIMON study. Pain.

[16] De Miquel C.A., Campayo J.G., Flórez M.T., Arguelles J.M.G., Tarrio E.B., Montoya M.G., Martin Á.P., Salio A.M., Fuentes J.V., Alberch E.A. (2011). Interdisciplinary consensus document for the treatment of fibromyalgia. Actas Esp. Psiquiatr..

[17] Häuser W., Bernardy K., Arnold B., Offenbächer M., Schiltenwolf M. (2009). Efficacy of multicomponent treatment in fibromyalgia syndrome: A meta-analysis of randomized controlled clinical trials. Arthritis Rheum..

[18] Macfarlane G.J., Kronisch C., Dean L.E., Atzeni F., Häuser W., Fluß E., Choy E., Kosek E., Amris K., Branco J. (2016). EULAR revised recommendations for the management of fibromyalgia. Ann. Rheum. Dis..

[19] Bernardy K., Klose P., Welsch P., Häuser W. (2018). Efficacy, acceptability and safety of cognitive behavioural therapies in fibromyalgia syndrome—A systematic review and meta-analysis of randomized controlled trials. Eur. J. Pain.

[20] Galan-Martin M.A., Montero-Cuadrado F., Lluch-Girbes E., Coca-López M.C., Mayo-Iscar A., Cuesta-Vargas A. (2020). Pain Neuroscience Education and Physical Exercise therapy for Patients with Chronic Spinal Pain in Spanish Physiotherapy Primary Care: A Pragmatic Randomized Controlled Trial. J. Clin. Med..

[21] Malfliet A., Van Oosterwijck J., Meeus M., Cagnie B., Danneels L., Dolphens M., Buyl R., Nijs J. (2017). Kinesiophobia and maladaptive coping strategies prevent improvements in pain catastrophizing following pain neuroscience education in fibromyalgia/chronic fatigue syndrome: An explorative study. Physiother. Theory Pract..

[22] McDowell C.P., Cook D.B., Herring M.P. (2017). The Effects of Exercise Training on Anxiety in Fibromyalgia Patients: A Meta-analysis. Med. Sci. Sports Exerc..

[23] Nishishinya M.B., Rivera J., Alegre C., Pereda C.A. (2016). Non-pharmacologic and alternative treatments in fibromyalgia. Med. Clin..

[24] Sharpe L., Jones E., Ashton-James C.E., Nicholas M.K., Refshauge K. (2020). Necessary components of psychological treatment in pain management programs: A Delphi study. Eur. J. Pain.

[25] Sosa-Reina M.D., Núñez-Nagy S., Gallego-Izquierdo T., Pecos-Martín D., Monserrat J., Álvarez-Mon M. (2017). Effectiveness of Exercise therapy in fibromyalgia syndrome: A systematic review and meta-analysis of randomized clinical trials. BioMed Res. Int..

[26] Pérez-Aranda A., D’Amico F., Feliu-Soler A., McCracken L.M., Peñarrubia-María M.T., Andrés-Rodríguez L., Luciano J.V. (2019). Cost–Utility of Mindfulness-Based Stress Reduction for Fibromyalgia versus a Multicomponent Intervention and Usual Care: A 12-Month Randomized Controlled Trial (EUDAIMON Study). J. Clin. Med..

[27] Serrat M., Sanabria-Mazo J.P., Musté M., Soler A.F., Méndez-Ulrich J.L., Sanz A., Luciano J.V., Almirall M. (2020). Effectiveness of a Multicomponent Treatment based on Pain Neuroscience Education, Therapeutic Exercise, Cognitive Behavioural Therapy, and Mindfulness in Patients with Fibromyalgia (FIBROWALK study): A Randomized Controlled Trial. PsyArXiv.

[28] Serrat M., Sanabria-Mazo J.P., García-Troiteiro E., Fontcuberta A., Mateo-Canedo C., Almirall M., Feliu-Soler A., Méndez-Ulrich J.L., Sanz A., Luciano J.V. (2020). Efficacy of a Multicomponent Intervention for Fibromyalgia Based on Pain Neuroscience Education, Exercise Therapy, Psychological Support, and Nature Exposure (NAT-FM): Study Protocol of a Randomized Controlled Trial. Int. J. Environ. Res. Public Health.

[29] Cole H., Triguero-Mas M., Connolly J.J., Anguelovski I. (2019). Determining the health benefits of green space: Does gentrification matter?. Health Place.

[30] Preuß M., Nieuwenhuijsen M., Márquez S., Cirach M., Dadvand P., Triguero-Mas M., Gidlow C., Grazuleviciene R., Kruize H., Zijlema W.L. (2019). Low Childhood Nature Exposure is Associated with Worse Mental Health in Adulthood. Int. J. Environ. Res. Public Health.

[31] Trøstrup C.H., Christiansen A.B., Stølen K.S., Nielsen P.K., Stelter R. (2019). The effect of nature exposure on the mental health of patients: A systematic review. Qual. Life Res..

[32] Zijlema W.L., Avila-Palencia I., Triguero-Mas M., Gidlow C., Maas J., Kruize H., Andrusaityte S., Grazuleviciene R., Nieuwenhuijsen M.J. (2018). Active commuting through natural environments is associated with better mental health: Results from the PHENOTYPE project. Environ. Int..

[33] Berman M.G., Kross E., Krpan K.M., Askren M.K., Burson A., Deldin P.J., Kaplan S., Sherdell L., Gotlib I.H., Jonides J. (2012). Interacting with nature improves cognition and affect for individuals with depression. J. Affect. Disord..

[34] Luttenberger K., Stelzer E.-M., Först S., Schopper M., Kornhuber J., Book S. (2015). Indoor rock climbing (bouldering) as a new treatment for depression: Study design of a waitlist-controlled randomized group pilot study and the first results. BMC Psychiatry.

[35] Stanhope J., Breed M.F., Weinstein P. (2020). Exposure to greenspaces could reduce the high global burden of pain. Environ. Res..

[36] López-Pousa S., Bassets Pagès G., Monserrat-Vila S., Blanco M.D.G., Colomé J.H., Garre-Olmo J. (2015). Sense of Well-Being in Patients with Fibromyalgia: Aerobic Exercise Program in a Mature Forest—A Pilot Study. Evid.-Based Complement. Altern. Med..

[37] Hofmann S.G., Hayes S.C. (2019). The Future of Intervention Science: Process-Based Therapy. Clin. Psychol. Sci..

[38] Sanabria-Mazo J.P., Serrat M., Canedo C.M., Soler A.F., Almirall M., Méndez-Ulrich J.L., Luciano J.V., Sanz A. (2020). Proof of Concept of a Treatment for Fibromyalgia Based on Physical Activity, Psychological Support, and Exposure to Nature (NAT-FM). PsyArXiv..

[39] Moher D., Hopewell S., Schulz K.F., Montori V., Gøtzsche P.C., Devereaux P.J., Elbourne D., Egger M., Altman D.G. (2010). CONSORT 2010 Statement: Updated guidelines for reporting parallel group randomized trials. BMJ.

[40] Wolfe F., Smythe H.A., Yunus M.B., Bennett R.M., Bombardier C., Goldenberg D.L., Tugwell P., Campbell S.M., Abeles M., Clark P. (1990). The american college of rheumatology. Criteria for the classification of fibromyalgia. Arthritis Rheum..

[41] Wolfe F., Clauw D.J., Fitzcharles M.-A., Goldenberg D.L., Häuser W., Katz R.S., Mease P.J., Russell A.S., Russell I.J., Winfield J.B. (2011). Fibromyalgia Criteria and Severity Scales for Clinical and Epidemiological Studies: A Modification of the ACR Preliminary Diagnostic Criteria for Fibromyalgia. J. Rheumatol..

[42] Segura-Jiménez V., Aparicio V.A., Álvarez-Gallardo I.C., Soriano-Maldonado A., Estévez-López F., Delgado-Fernández M., Carbonell-Baeza A. (2014). Validation of the modified 2010 American College of Rheumatology diagnostic criteria for fibromyalgia in a Spanish population. Rheumatology (Oxford).

[43] Bennett R.M., Friend R., Jones K.D., Ward R., Han B.K., Ross R.L. (2009). The Revised Fibromyalgia Impact Questionnaire (FIQR): Validation and psychometric properties. Arthritis Res. Ther..

[44] Luciano J.V., Aguado J., Serrano-Blanco A., Calandre E.P., Rodriguez-Lopez C.M. (2013). Dimensionality, reliability, and validity of the Revised Fibromyalgia Impact Questionnaire in two Spanish samples. Arthritis Care Res..

[45] Esteve-Vives J., Redondo J.R., Salvat M.I.S., Blanco M.D.G., De Miquel C.A. (2007). Propuesta de una versión de consenso del Fibromyalgia Impact Questionnaire (FIQ) para la población española. Reumatol. Clin..

[46] Salgueiro M., García-Leiva J.M., Ballesteros J., Hidalgo J., Molina R., Calandre E.P. (2013). Validation of a Spanish version of the Revised Fibromyalgia Impact Questionnaire (FIQR). Health Qual. Life Outcomes.

[47] Zigmond A.S., Snaith R.P. (1983). The Hospital Anxiety and Depression Scale. Acta Psychiatr. Scand..

[48] Herrero M.J., Blanch J., Peri J.M., De Pablo J., Pintor L., Bulbena A. (2003). A validation study of the hospital anxiety and depression scale (HADS) in a Spanish population. Gen. Hosp. Psychiatry.

[49] Ware J.E., Sherbourne C.D. (1992). The MOS 36-item short-form health survey (SF-36) (I). Conceptual framework and item selection. Med. Care.

[50] Alonso J., Prieto L., Antó J.M. (1995). The Spanish version of the SF-36 Health Survey (the SF-36 health questionnaire): An instrument for measuring clinical results. Med. Clin. (Barc.).

[51] Watson D., Clark L.A., Tellegen A. (1998). Development and validation of brief measures of positive and negative affect: The PANAS Scales. J. Person. Soc. Psych..

[52] López I., Hervás G., Vázquez C. (2015). Adaptación de la “Escala de afecto positivo y negativo” (PANAS) en una muestra general española. Behav. Psychol..

[53] Rosenberg M. (1965). Society and the Adolescent Self-Image.

[54] Vázquez A.J., Vázquez R. (2004). Escala de autoestima de Rosenberg: Fiabilidad y validez en población clínica española. Apuntes Psicol..

[55] Cohen S., Kamarck T., Mermelstein R. (1983). A Global Measure of Perceived Stress. J. Health Soc. Behav..

[56] Pedrero E.J., Ruiz J.M., Lozoya P., Rojo G., Llanero M., Puerta C. (2015). La “Escala de Estrés Percibido”: Estudio psicométrico sin restricciones en población no clínica y adictos a sustancias en tratamiento. Behav. Psychol..

[57] Kori S.H., Miller R.P., Todd D.D. (1990). Kinesiophobia: A new view of chronic pain behavior. Pain Manag..

[58] Gómez-Pérez L., López-Martínez A.E., Ruíz-Párraga G.T. (2011). Psychometric Properties of the Spanish Version of the Tampa Scale for Kinesiophobia (TSK). J. Pain.

[59] Sullivan M.J.L., Bishop S., Pivik J. (1995). The Pain Catastrophizing Scale: Development and validation. Psychol. Assess..

[60] García J., Rodero B., Alda M., Sobradiela N., Montero J., Moreno S. (2008). Validación de la versión española de la escala de la catastrofización ante el dolor (Pain Catastrophizing Scale) en la fibromialgia. Med. Clín..

[61] Wallston K.A. (1992). Hocus-pocus, the focus isn’t strictly on locus: Rotter’s social learning theory modified for health. Cogn. Ther. Res..

[62] Fernández J., Doval E., Blasco T., Álvarez M., Sanz A. (1998). Validación de la Escala de Competencia Personal de Wallston: Implicaciones para el estudio del estrés. Ans. Est..

[63] Garnefski N., Kraaij V., Spinhoven P. (2001). Negative life events, cognitive emotion regulation and emotional problems. Pers. Individ. Differ..

[64] Feliu-Soler A., Reche-Camba E., Borràs X., Pérez-Aranda A., Andrés-Rodríguez L., Peñarrubia-María M.T., Navarro-Gil M., García-Campayo J., Bellón J.A., Luciano J.V. (2017). Psychometric Properties of the Cognitive Emotion Regulation Questionnaire (CERQ) in Patients with Fibromyalgia Syndrome. Front. Psychol..

[65] Shiffman S., Stone A.A., Hufford M.R. (2008). Ecological Momentary assessment. Annu. Rev. Clin. Psychol..

[66] Csikszentmihalyi M. (2014). Validity and Reliability of the Experience-Sampling Method.

[67] May M., Junghaenel D.U., Ono M., Stone A.A., Schneider S. (2018). Ecological Momentary Assessment Methodology in Chronic Pain Research: A Systematic Review. J. Pain.

[68] Twisk J., De Boer M., De Vente W., Heymans M. (2013). Multiple imputation of missing values was not necessary before performing a longitudinal mixed-model analysis. J. Clin. Epidemiol..

[69] Morris S.B. (2008). Estimating Effect Sizes From Pretest-Posttest-Control Group Designs. Organ. Res. Methods.

[70] Ollevier A., Vanneuville I., Carron P., Baetens T., Goderis T., Gabriel L., Van De Velde D. (2019). A 12-week multicomponent therapy in fibromyalgia improves health but not in concomitant moderate depression, an exploratory pilot study. Disabil. Rehabil..

