# Supplementary material for: Effectiveness of a Multicomponent Treatment for Fibromyalgia Based on Pain Neuroscience Education, Exercise Therapy, Psychological Support, and Nature Exposure (NAT-FM): A Pragmatic Randomized Controlled Trial
Source: J Clin Med. 2020 Oct 18;9(10):3348. doi: 10.3390/jcm9103348 (PMC7603188; doi:10.3390/jcm9103348)
Supplement: Supplementary file 1 [file jcm-09-03348-s001.pdf]

Table S1. Correlation table between pre-6 week changes in process variables (TSK, PCS, PPCS and CERQ) and pre-post changes in main variables.

	FIQR	VAS-P	VAS-F	HADS-A	HADS-D	SF-36	SS	PANAS +	PANAS −	RSES
TSK	0.51 **	0.46 **	0.43 **	0.39 **	−0.41 **	−0.42 **	0.12	−0.21 *	0.05	0.07
PCS	0.42 **	0.36 **	0.32 **	0.32 **	0.35 **	−0.41 **	0.18	−0.09	0.16	0.13
PPCS	−0.46 **	−0.36 **	−0.14	−0.51 **	−0.49 **	0.43 **	−0.10	0.14	−0.22 *	−0.15
CERQ Acceptance	−0.15	−0.12	0.06	−0.29 *	−0.21 *	0.20 *	0.01	0.08	0.02	−0.01
CERQ Refocusing	0-.34 **	−0.20 *	−0.02	−0.35 **	−0.37 **	0.33 **	−0.18	0.08	−0.08	−0.00
CERQ Planning	−0.25 *	-0.16	−0.19	−0.19	−0.24 *	0.18	0.08	0.06	−0.01	−0.04
CERQ Positive reappraisal	−0.36 **	−0.29 *	−0.28 *	−0.38 **	−0.37 **	0.37 **	−0.08	−0.30 *	0.07	−0.23 *
CERQ Catastrophizing	0.17	0.06	0.03	0.15	0.09	−0.26 *	0.07	0.15	0.26 *	−0.01

TSK, Tampa scale for kinesiophobia; PCS, pain catastrophizing scale; PPCS, personal perceived competence scale; CERQ, cognitive emotion regulation questionnaire; FIQR, fibromyalgia impact questionnaire; VAS-P, visual analog scale for pain; VAS-F, visual analog scale for fatigue; HADS, hospital anxiety and depression scale—depression; SF36, short form survey (physical functioning subscale); PANAS +, positive affect and negative affect schedule—positive affect; PANAS −, positive affect and negative affect schedule—negative affect; RSES, Rosenberg self-esteem scale. Note: only process variables which experienced a significant pretreatment to six weeks change were included. Process measures finally included in mediational models can be identified from the gray background. * Means $p < 0.05$; ** means $p \leq 0.001$.
